# Supplementary material for: Use of the Thyromental Height Test for Prediction of Difficult Laryngoscopy: A Systematic Review and Meta-Analysis
Source: J Clin Med. 2022 Aug 21;11(16):4906. doi: 10.3390/jcm11164906 (PMC9409656; doi:10.3390/jcm11164906)
Supplement: Supplementary file 1 [file jcm-11-04906-s001.zip › Supplement Figures S1-S6.pdf]

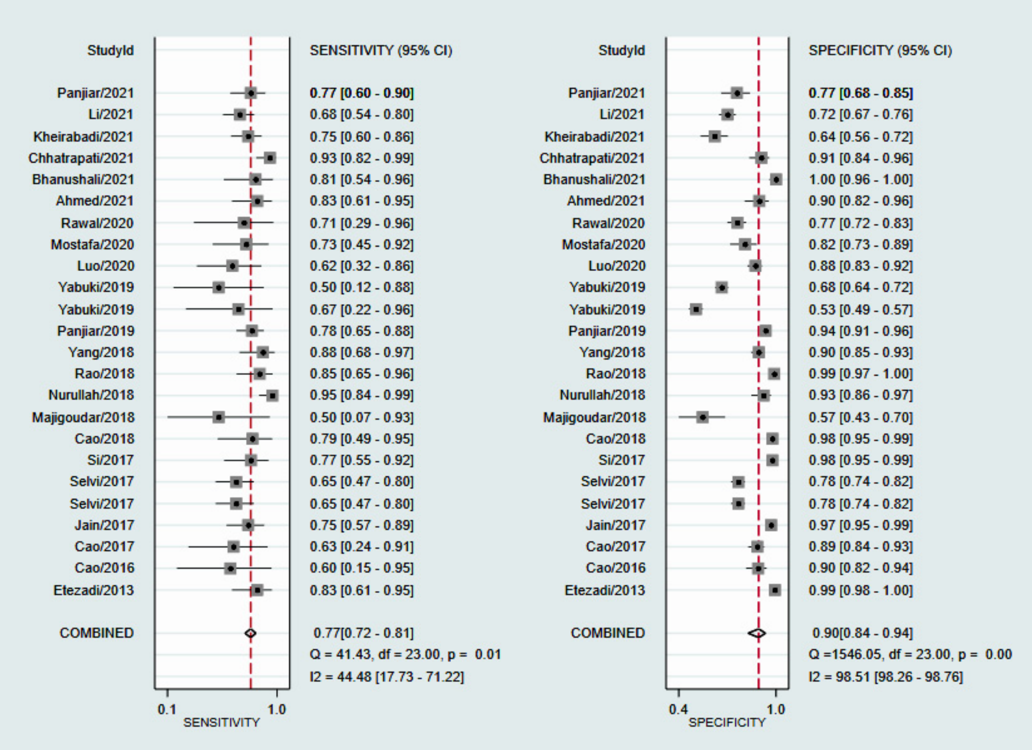

Supplement Figure S1 Forest plots of the analysis about the prediction value of TMHT for DL in terms of sensitivity and specificity after removing heterogeneous studies.

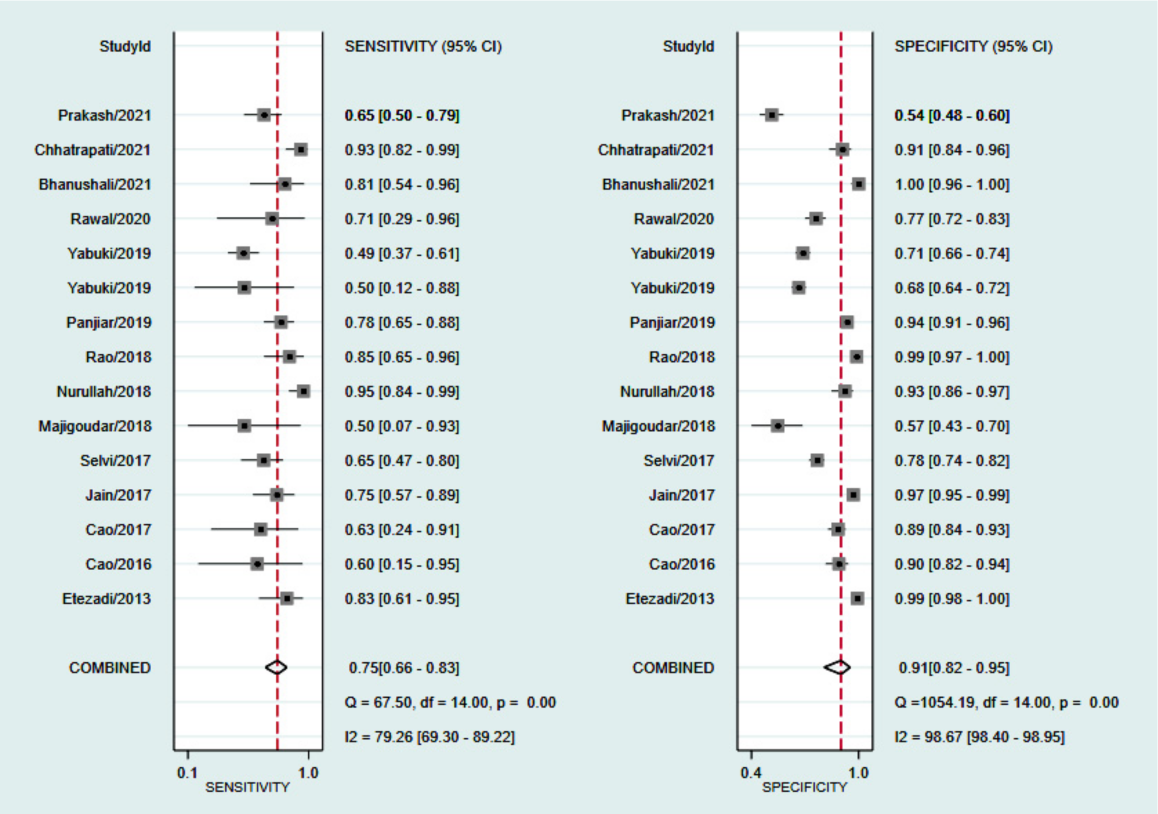

Supplement Figure S2 Forest plots of the analysis about the prediction value of TMHT for DL in terms of sensitivity and specificity with the data of only studies consisting of a 5 cm threshold.

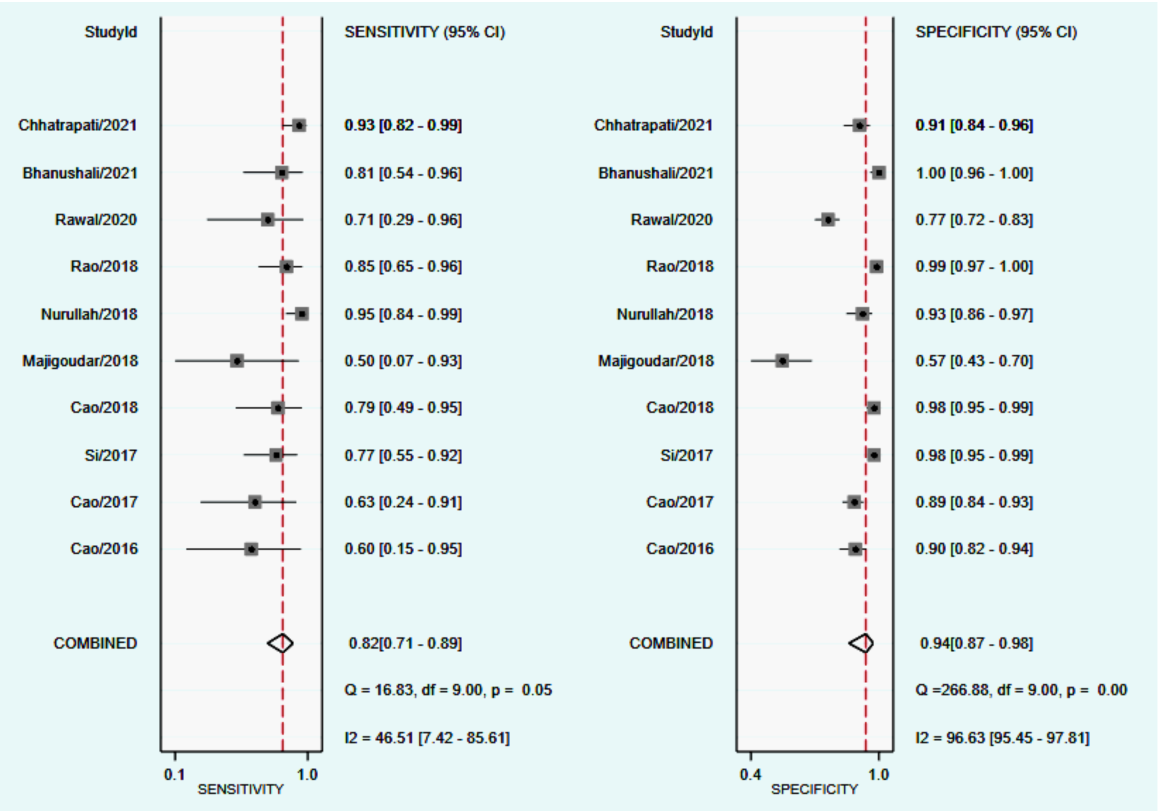

Supplement Figure S3 Forest plots of the analysis about the prediction value of TMHT for DL in terms of sensitivity and specificity with the data of only studies consisting of a prespecified threshold

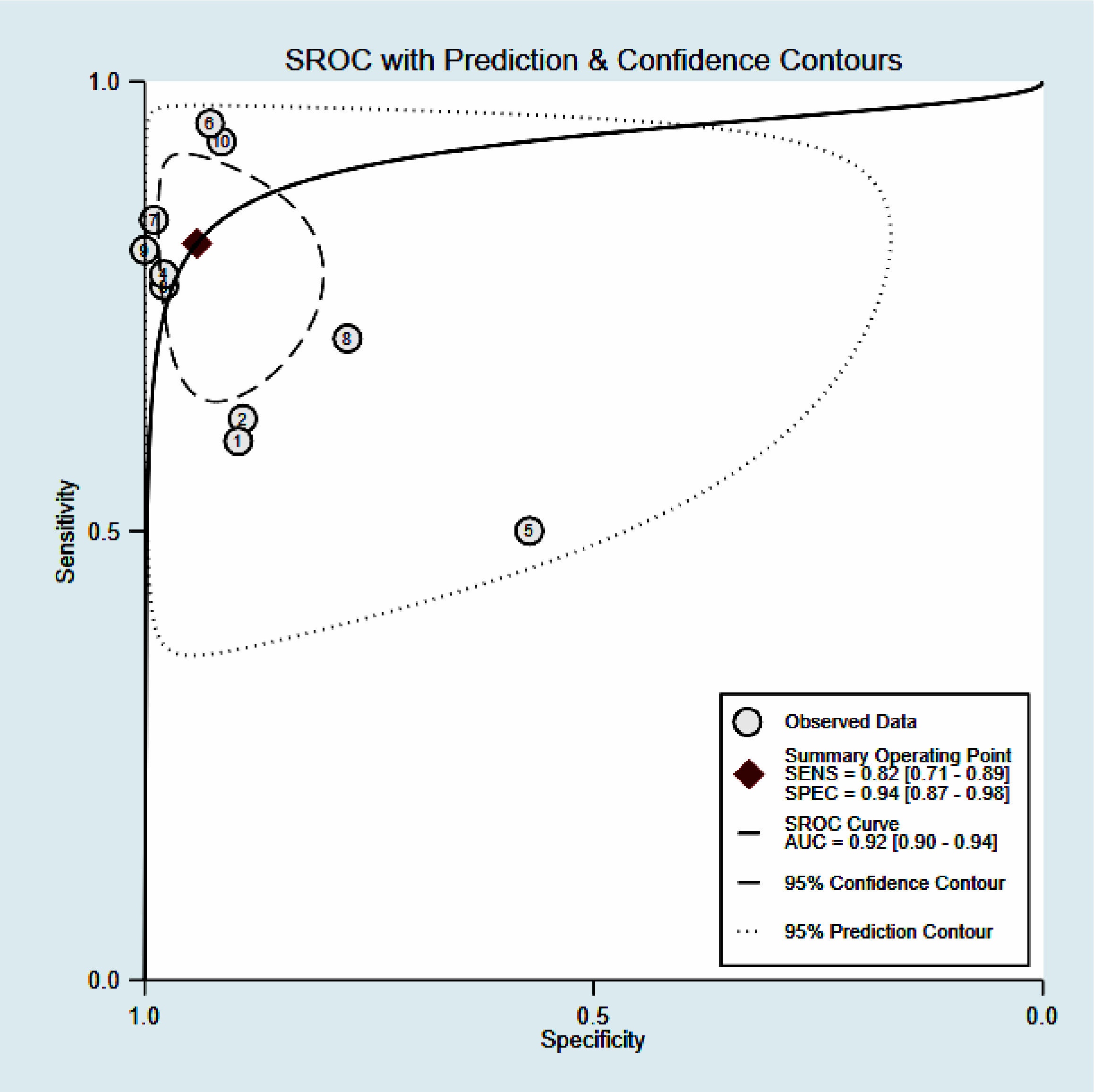

Supplement Figure S4 SROC for sensitivity and specificity of TMHT for prediction of DL with the data of studies consisting of a prespecified threshold.

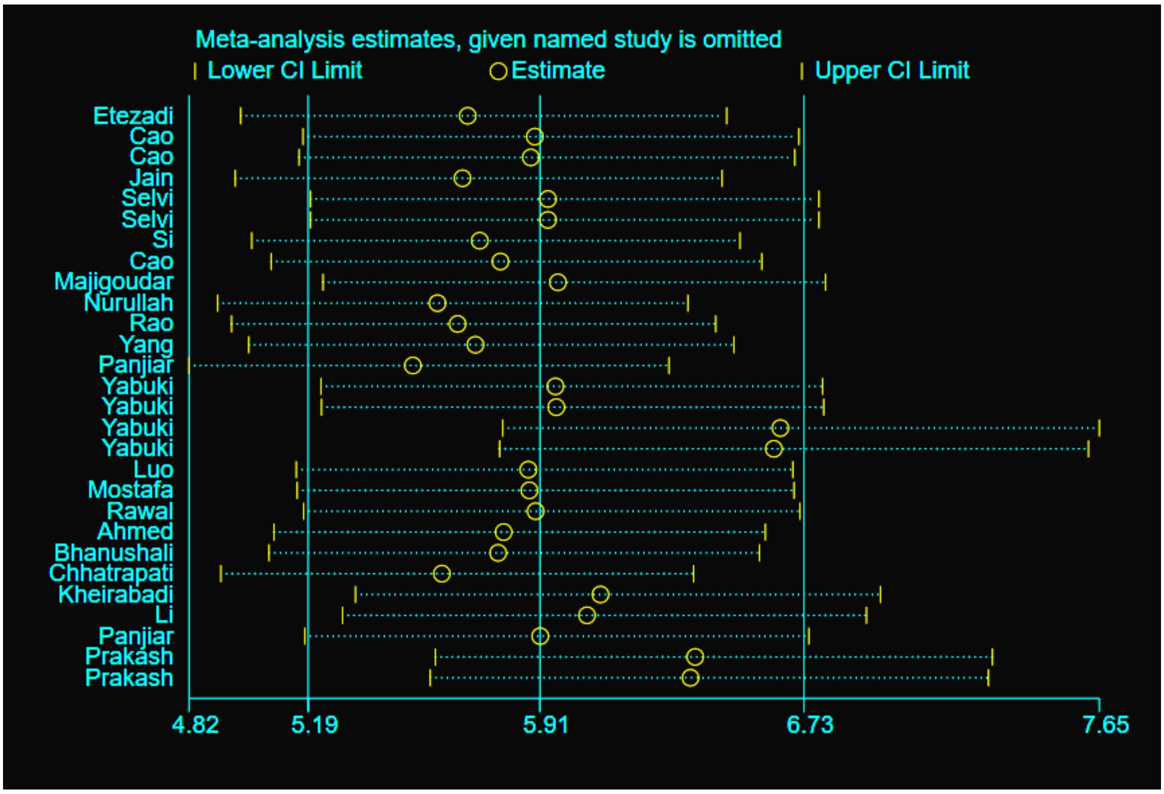

Supplement Figure S5 Forest plot of sensitivity analysis of all studies selected from literature review.

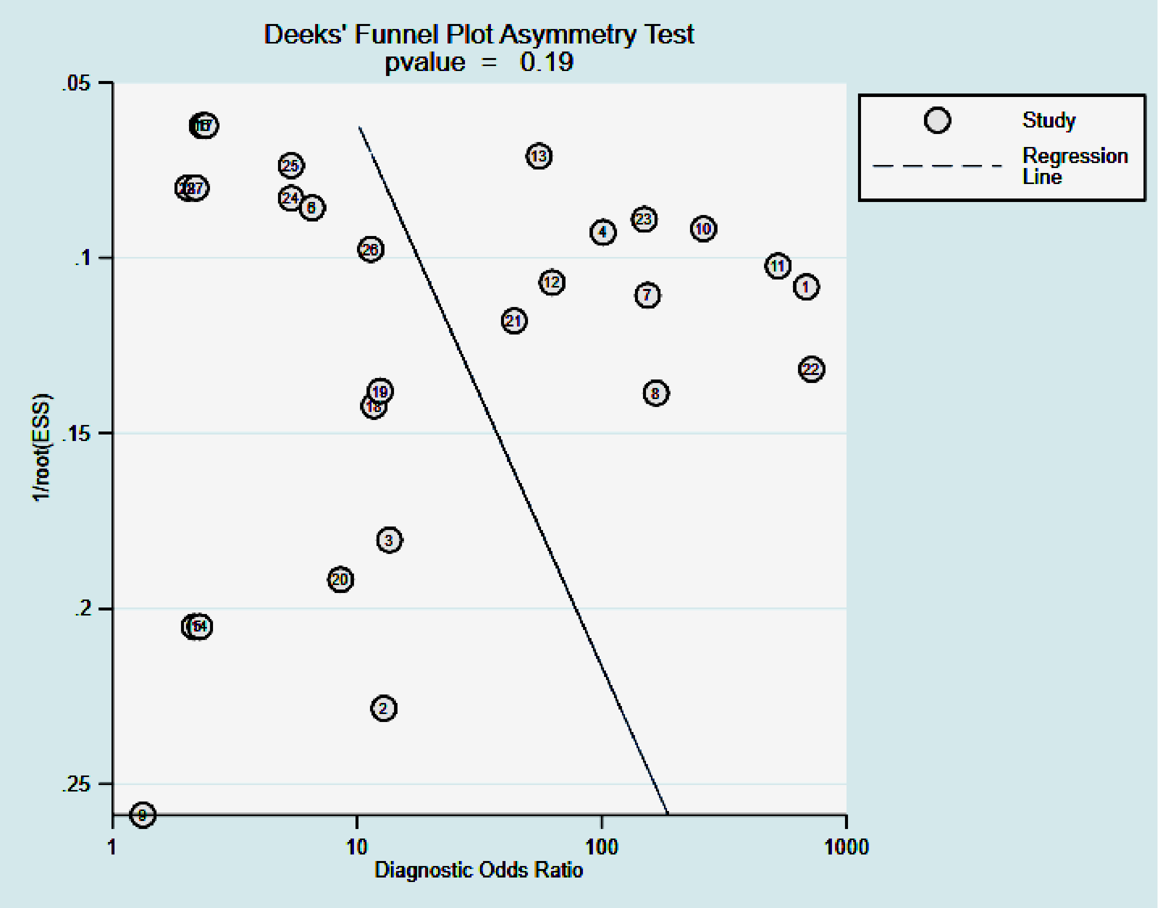

Supplement Figure S6 Deek's funnel plot asymmetry test for publication bias of all studies selected from literature review.
