# Supplementary material for: Use of the Thyromental Height Test for Prediction of Difficult Laryngoscopy: A Systematic Review and Meta-Analysis
Source: J Clin Med. 2022 Aug 21;11(16):4906. doi: 10.3390/jcm11164906 (PMC9409656; doi:10.3390/jcm11164906)
Supplement: Supplementary file 1 [file jcm-11-04906-s001.zip › Supplement Table S1 PubMed search strategy.pdf]

Supplement Table S1: PubMed search strategy

Search number	Query	Results
1	laryngoscopy[MeSH]	13,589
2	(((((((((Laryngoscopies[Title/Abstract]) OR (Laryngoscopic Surgical Procedures[Title/Abstract])) OR (Laryngoscopic Surgical Procedure[Title/Abstract])) OR (Procedure, Laryngoscopic Surgical[Title/Abstract])) OR (Procedures, Laryngoscopic Surgical[Title/Abstract])) OR (Surgical Procedure, Laryngoscopic[Title/Abstract])) OR (Surgery, Laryngoscopic[Title/Abstract])) OR (Surgical Procedures, Laryngoscopic[Title/Abstract])) OR (Laryngoscopic Surgery[Title/Abstract])) OR (Laryngoscopic Surgeries[Title/Abstract])) OR (Surgeries, Laryngoscopic[Title/Abstract])	1,587
3	#1 OR #2	14,263
4	Intubation[MeSH]	56,329
5	Intubations[Title/Abstract]	2,801
6	#4 OR #5	57,268
7	endotracheal intubation[MeSH]	41,406
8	((((((Intratracheal Intubation[Title/Abstract]) OR (Intratracheal Intubations[Title/Abstract])) OR (Intubations, Intratracheal[Title/Abstract])) OR (Intubation, Endotracheal[Title/Abstract])) OR (Endotracheal Intubation[Title/Abstract])) OR (Endotracheal Intubations[Title/Abstract])) OR (Intubations, Endotracheal[Title/Abstract])	19,057
9	#7 OR #8	48,337
10	airway management[MeSH]	126,797
11	((Management, Airway[Title/Abstract]) OR (Airway Control[Title/Abstract])) OR (Control, Airway[Title/Abstract])	840
12	#10 OR #11	127,272
13	#3 OR #6 OR #9 OR #12	156,747
14	Thyromental Height[MeSH]	8
15	(((Thyromental Height[Title/Abstract]) OR (Thyromental Height Test[Title/Abstract])) OR (TMH[Title/Abstract])) OR (TMHT[Title/Abstract])	788
16	#14 OR #15	796
17	#13 AND #16	23
