# Supplementary material for: Use of the Thyromental Height Test for Prediction of Difficult Laryngoscopy: A Systematic Review and Meta-Analysis
Source: J Clin Med. 2022 Aug 21;11(16):4906. doi: 10.3390/jcm11164906 (PMC9409656; doi:10.3390/jcm11164906)
Supplement: Supplementary file 1 [file jcm-11-04906-s001.zip › Supplement Table S2 Stata code used for meta-analysis.pdf]

Supplement Table S2: Stata code used for meta-analysis

Meta analysis	Stata code
Threshold effect and summary estimates	midas tp fp fn tn, res(all)
Forest plots and heterogeneity	midas tp fp fn tn, texts(0.7) bfor(dss) id(author year) ford fors<br>midas tp fp fn tn, texts(0.7) bfor(dlr) id(author year) ford fors<br>midas tp fp fn tn, texts(0.7) ufor(dlor) id(author year) ford fors
Sensitivity analysis	metaninf tp fp fn tn, lable(namevar=author,year=year)
Publication bias	midas tp fp fn tn, pubbias
SROC curve	midas tp fp fn tn, plot sroc(both)
